# Methodology or method? A critical review of qualitative case study reports

**DOI:** 10.3402/qhw.v9.23606

**Published:** 2014-05-07

**Authors:** Nerida Hyett, Amanda Kenny, Virginia Dickson-Swift

**Affiliations:** Faculty of Health Sciences, La Trobe Rural Health School, La Trobe University, Bendigo, Australia

**Keywords:** Case studies, health research, research design, interdisciplinary research, qualitative research, literature review

## Abstract

Despite on-going debate about credibility, and reported limitations in comparison to other approaches, case study is an increasingly popular approach among qualitative researchers. We critically analysed the methodological descriptions of published case studies. Three high-impact qualitative methods journals were searched to locate case studies published in the past 5 years; 34 were selected for analysis. Articles were categorized as health and health services (*n=*12), social sciences and anthropology (*n=*7), or methods (*n=*15) case studies. The articles were reviewed using an adapted version of established criteria to determine whether adequate methodological justification was present, and if study aims, methods, and reported findings were consistent with a qualitative case study approach. Findings were grouped into five themes outlining key methodological issues: case study methodology or method, case of something particular and case selection, contextually bound case study, researcher and case interactions and triangulation, and study design inconsistent with methodology reported. Improved reporting of case studies by qualitative researchers will advance the methodology for the benefit of researchers and practitioners.

Case study research is an increasingly popular approach among qualitative researchers (Thomas, 2011). Several prominent authors have contributed to methodological developments, which has increased the popularity of case study approaches across disciplines (Creswell, 2013b; Denzin & Lincoln, 2011b; Merriam, 2009; Ragin & Becker, 1992; Stake, 1995; Yin, 2009). Current qualitative case study approaches are shaped by paradigm, study design, and selection of methods, and, as a result, case studies in the published literature vary. Differences between published case studies can make it difficult for researchers to define and understand case study as a methodology.

Experienced qualitative researchers have identified case study research as a stand-alone qualitative approach (Denzin & Lincoln, 2011b). Case study research has a level of flexibility that is not readily offered by other qualitative approaches such as grounded theory or phenomenology. Case studies are designed to suit the case and research question and published case studies demonstrate wide diversity in study design. There are two popular case study approaches in qualitative research. The first, proposed by Stake (1995) and Merriam (2009), is situated in a social constructivist paradigm, whereas the second, by Yin (2012), Flyvbjerg (2011), and Eisenhardt (1989), approaches case study from a post-positivist viewpoint. Scholarship from both schools of inquiry has contributed to the popularity of case study and development of theoretical frameworks and principles that characterize the methodology.

The diversity of case studies reported in the published literature, and on-going debates about credibility and the use of case study in qualitative research practice, suggests that differences in perspectives on case study methodology may prevent researchers from developing a mutual understanding of practice and rigour. In addition, discussion about case study limitations has led some authors to query whether case study is indeed a methodology (Luck, Jackson, & Usher, 2006; Meyer, 2001; Thomas, 2010; Tight, 2010). Methodological discussion of qualitative case study research is timely, and a review is required to analyse and understand how this methodology is applied in the qualitative research literature. The aims of this study were to review methodological descriptions of published qualitative case studies, to review how the case study methodological approach was applied, and to identify issues that need to be addressed by researchers, editors, and reviewers. An outline of the current definitions of case study and an overview of the issues proposed in the qualitative methodological literature are provided to set the scene for the review.

## Definitions of qualitative case study research

Case study research is an investigation and analysis of a single or collective case, intended to capture the complexity of the object of study (Stake, 1995). Qualitative case study research, as described by Stake (1995), draws together “naturalistic, holistic, ethnographic, phenomenological, and biographic research methods” in a bricoleur design, or in his words, “a palette of methods” (Stake, 1995, pp. xi–xii). Case study methodology maintains deep connections to core values and intentions and is “particularistic, descriptive and heuristic” (Merriam, 2009, p. 46).

As a study design, case study is defined by interest in individual cases rather than the methods of inquiry used. The selection of methods is informed by researcher and case intuition and makes use of naturally occurring sources of knowledge, such as people or observations of interactions that occur in the physical space (Stake, 1998). Thomas (2011) suggested that “analytical eclecticism” is a defining factor (p. 512). Multiple data collection and analysis methods are adopted to further develop and understand the case, shaped by context and emergent data (Stake, 1995). This qualitative approach “explores a real-life, contemporary bounded system (a ***case***) or multiple bounded systems (cases) over time, through detailed, in-depth data collection involving ***multiple sources of information*** … and reports a ***case description*** **and** ***case themes***” (Creswell, 2013b, p. 97). Case study research has been defined by the unit of analysis, the process of study, and the outcome or end product, all essentially the case (Merriam, 2009).

The case is an object to be studied for an identified reason that is peculiar or particular. Classification of the case and case selection procedures informs development of the study design and clarifies the research question. Stake (1995) proposed three types of cases and study design frameworks. These include the intrinsic case, the instrumental case, and the collective instrumental case. The intrinsic case is used to understand the particulars of a single case, rather than what it represents. An instrumental case study provides insight on an issue or is used to refine theory. The case is selected to advance understanding of the object of interest. A collective refers to an instrumental case which is studied as multiple, nested cases, observed in unison, parallel, or sequential order. More than one case can be simultaneously studied; however, each case study is a concentrated, single inquiry, studied holistically in its own entirety (Stake, 1995, 1998).

Researchers who use case study are urged to seek out what is common and what is particular about the case. This involves careful and in-depth consideration of the nature of the case, historical background, physical setting, and other institutional and political contextual factors (Stake, 1998). An interpretive or social constructivist approach to qualitative case study research supports a transactional method of inquiry, where the researcher has a personal interaction with the case. The case is developed in a relationship between the researcher and informants, and presented to engage the reader, inviting them to join in this interaction and in case discovery (Stake, 1995). A postpositivist approach to case study involves developing a clear case study protocol with careful consideration of validity and potential bias, which might involve an exploratory or pilot phase, and ensures that all elements of the case are measured and adequately described (Yin, 2009, 2012).

## Current methodological issues in qualitative case study research

The future of qualitative research will be influenced and constructed by the way research is conducted, and by what is reviewed and published in academic journals (Morse, 2011). If case study research is to further develop as a principal qualitative methodological approach, and make a valued contribution to the field of qualitative inquiry, issues related to methodological credibility must be considered. Researchers are required to demonstrate rigour through adequate descriptions of methodological foundations. Case studies published without sufficient detail for the reader to understand the study design, and without rationale for key methodological decisions, may lead to research being interpreted as lacking in quality or credibility (Hallberg, 2013; Morse, 2011).

There is a level of artistic license that is embraced by qualitative researchers and distinguishes practice, which nurtures creativity, innovation, and reflexivity (Denzin & Lincoln, 2011b; Morse, 2009). Qualitative research is “inherently multimethod” (Denzin & Lincoln, 2011a, p. 5); however, with this creative freedom, it is important for researchers to provide adequate description for methodological justification (Meyer, 2001). This includes paradigm and theoretical perspectives that have influenced study design. Without adequate description, study design might not be understood by the reader, and can appear to be dishonest or inaccurate. Reviewers and readers might be confused by the inconsistent or inappropriate terms used to describe case study research approach and methods, and be distracted from important study findings (Sandelowski, 2000). This issue extends beyond case study research, and others have noted inconsistencies in reporting of methodology and method by qualitative researchers. Sandelowski (2000, 2010) argued for accurate identification of qualitative description as a research approach. She recommended that the selected methodology should be harmonious with the study design, and be reflected in methods and analysis techniques. Similarly, Webb and Kevern (2000) uncovered inconsistencies in qualitative nursing research with focus group methods, recommending that methodological procedures must cite seminal authors and be applied with respect to the selected theoretical framework. Incorrect labelling using case study might stem from the flexibility in case study design and non-directional character relative to other approaches (Rosenberg & Yates, 2007). Methodological integrity is required in design of qualitative studies, including case study, to ensure study rigour and to enhance credibility of the field (Morse, 2011).

Case study has been unnecessarily devalued by comparisons with statistical methods (Eisenhardt, 1989; Flyvbjerg, 2006, 2011; Jensen & Rodgers, 2001; Piekkari, Welch, & Paavilainen, 2009; Tight, 2010; Yin, 1999). It is reputed to be the “the weak sibling” in comparison to other, more rigorous, approaches (Yin, 2009, p. xiii). Case study is not an inherently comparative approach to research. The objective is not statistical research, and the aim is not to produce outcomes that are generalizable to all populations (Thomas, 2011). Comparisons between case study and statistical research do little to advance this qualitative approach, and fail to recognize its inherent value, which can be better understood from the interpretive or social constructionist viewpoint of other authors (Merriam, 2009; Stake, 1995). Building on discussions relating to “fuzzy” (Bassey, 2001), or naturalistic generalizations (Stake, 1978), or transference of concepts and theories (Ayres, Kavanaugh, & Knafl, 2003; Morse et al., 2011) would have more relevance.

Case study research has been used as a catch-all design to justify or add weight to fundamental qualitative descriptive studies that do not fit with other traditional frameworks (Merriam, 2009). A case study has been a “convenient label for our research—when we ‘can't think of anything ‘better”—in an attempt to give it [qualitative methodology] some added respectability” (Tight, 2010, p. 337). Qualitative case study research is a pliable approach (Merriam, 2009; Meyer, 2001; Stake, 1995), and has been likened to a “curious methodological limbo” (Gerring, 2004, p. 341) or “paradigmatic bridge” (Luck et al., 2006, p. 104), that is on the borderline between postpositivist and constructionist interpretations. This has resulted in inconsistency in application, which indicates that flexibility comes with limitations (Meyer, 2001), and the open nature of case study research might be off-putting to novice researchers (Thomas, 2011). The development of a well-(in)formed theoretical framework to guide a case study should improve consistency, rigour, and trust in studies published in qualitative research journals (Meyer, 2001).

## Methods

### Assessment of rigour

The purpose of this study was to analyse the methodological descriptions of case studies published in qualitative methods journals. To do this we needed to develop a suitable framework, which used existing, established criteria for appraising qualitative case study research rigour (Creswell, 2013b; Merriam, 2009; Stake, 1995). A number of qualitative authors have developed concepts and criteria that are used to determine whether a study is rigorous (Denzin & Lincoln, 2011b; Lincoln, 1995; Sandelowski & Barroso, 2002). The criteria proposed by Stake (1995) provide a framework for readers and reviewers to make judgements regarding case study quality, and identify key characteristics essential for good methodological rigour. Although each of the factors listed in Stake's criteria could enhance the quality of a qualitative research report, in Table I we present an adapted criteria used in this study, which integrates more recent work by Merriam (2009) and Creswell (2013b). Stake's (1995) original criteria were separated into two categories. The first list of general criteria is “relevant for all qualitative research.” The second list, “high relevance to qualitative case study research,” was the criteria that we decided had higher relevance to case study research. This second list was the main criteria used to assess the methodological descriptions of the case studies reviewed. The complete table has been preserved so that the reader can determine how the original criteria were adapted.

**Table I:** Framework for assessing quality in qualitative case study research.

Checklist for assessing the quality of a case study report
Relevant for all qualitative research
1. Is this report easy to read?
2. Does it fit together, each sentence contributing to the whole?
3. Does this report have a conceptual structure (i.e., themes or issues)?
4. Are its issues developed in a series and scholarly way?
5. Have quotations been used effectively?
6. Has the writer made sound assertions, neither over- or under-interpreting?
7. Are headings, figures, artefacts, appendices, indexes effectively used?
8. Was it edited well, then again with a last minute polish?
9. Were sufficient raw data presented?
10. Is the nature of the intended audience apparent?
11. Does it appear that individuals were put at risk?
High relevance to qualitative case study research
12. Is the case adequately defined?
13. Is there a sense of story to the presentation?
14. Is the reader provided some vicarious experience?
15. Has adequate attention been paid to various contexts?
16. Were data sources well-chosen and in sufficient number?
17. Do observations and interpretations appear to have been triangulated?
18. Is the role and point of view of the researcher nicely apparent?
19. Is empathy shown for all sides?
20. Are personal intentions examined?
Added from Merriam (2009)
21. Is the case study particular?
22. Is the case study descriptive?
23. Is the case study heuristic?
Added from Creswell (2013b)
24. Was study design appropriate to methodology?

Adapted from Stake (1995, p. 131).

### Study design

The critical review method described by Grant and Booth (2009) was used, which is appropriate for the assessment of research quality, and is used for literature analysis to inform research and practice. This type of review goes beyond the mapping and description of scoping or rapid reviews, to include “analysis and conceptual innovation” (Grant & Booth, 2009, p. 93). A critical review is used to develop existing, or produce new, hypotheses or models. This is different to systematic reviews that answer clinical questions. It is used to evaluate existing research and competing ideas, to provide a “launch pad” for conceptual development and “subsequent testing” (Grant & Booth, 2009, p. 93).

Qualitative methods journals were located by a search of the 2011 ISI Journal Citation Reports in Social Science, via the database Web of Knowledge (see m.webofknowledge.com). No “qualitative research methods” category existed in the citation reports; therefore, a search of all categories was performed using the term “qualitative.” In Table II, we present the qualitative methods journals located, ranked by impact factor. The highest ranked journals were selected for searching. We acknowledge that the impact factor ranking system might not be the best measure of journal quality (Cheek, Garnham, & Quan, 2006); however, this was the most appropriate and accessible method available.

**Table II:** International Journal of Qualitative Studies on Health and Well-being.

Journal title	2011 impact factor	5-year impact factor
*Qualitative Health Research*	2.188	2.432
*Qualitative Research*	1.426	N/A
*Qualitative Inquiry*	0.839	1.850
*Qualitative Sociology*	0.780	N/A
*International Journal of Qualitative Studies on Health and Wellbeing*	0.612	N/A

### Search strategy

In March 2013, searches of the journals, *Qualitative Health Research*, *Qualitative Research*, and *Qualitative Inquiry* were completed to retrieve studies with “case study” in the abstract field. The search was limited to the past 5 years (1 January 2008 to 1 March 2013). The objective was to locate published qualitative case studies suitable for assessment using the adapted criterion. Viewpoints, commentaries, and other article types were excluded from review. Title and abstracts of the 45 retrieved articles were read by the first author, who identified 34 empirical case studies for review. All authors reviewed the 34 studies to confirm selection and categorization. In Table III, we present the 34 case studies grouped by journal, and categorized by research topic, including health sciences, social sciences and anthropology, and methods research. There was a discrepancy in categorization of one article on pedagogy and a new teaching method published in *Qualitative Inquiry* (Jorrín-Abellán, Rubia-Avi, Anguita-Martínez, Gómez-Sánchez, & Martínez-Mones, 2008). Consensus was to allocate to the methods category.

**Table III:** Outcomes of search of qualitative methods journals.

Journal title	Date of search	Number of studies located	Number of full text studies extracted	Health sciences	Social sciences and anthropology	Methods
*Qualitative Health Research*	4 Mar 2013	18	16	Barone (2010); Bronken et al. (2012); Colón-Emeric et al. (2010); Fourie and Theron (2012); Gallagher et al. (2013); Gillard et al. (2011); Hooghe et al. (2012); Jackson et al. (2012); Ledderer (2011); Mawn et al. (2010); Roscigno et al. (2012); Rytterström et al. (2013)	Nil	Austin, Park, and Goble (2008); Broyles, Rodriguez, Price, Bayliss, and Sevick (2011); De Haene et al. (2010); Fincham et al. (2008)
*Qualitative Research*	7 Mar 2013	11	7	Nil	Adamson and Holloway (2012); Coltart and Henwood (2012)	Buckley and Waring (2013); Cunsolo Willox et al. (2013); Edwards and Weller (2012); Gratton and O'Donnell (2011); Sumsion (2013)
*Qualitative Inquiry*	4 Mar 2013	16	11	Nil	Buzzanell and D’Enbeau (2009); D'Enbeau et al. (2010); Nagar-Ron and Motzafi-Haller (2011); Snyder-Young (2011); Yeh (2013)	Ajodhia-Andrews and Berman (2009); Alexander et al. (2012); Jorrín-Abellán et al. (2008); Nairn and Panelli (2009); Nespor (2012); Wimpenny and Savin-Baden (2012)
Total		45	34	12	7	15

In Table III, the number of studies located, and final numbers selected for review have been reported. *Qualitative Health Research* published the most empirical case studies (*n=*16). In the health category, there were 12 case studies of health conditions, health services, and health policy issues, all published in *Qualitative Health Research*. Seven case studies were categorized as social sciences and anthropology research, which combined case study with biography and ethnography methodologies. All three journals published case studies on methods research to illustrate a data collection or analysis technique, methodological procedure, or related issue.

## Findings

The methodological descriptions of 34 case studies were critically reviewed using the adapted criteria. All articles reviewed contained a description of study methods; however, the length, amount of detail, and position of the description in the article varied. Few studies provided an accurate description and rationale for using a qualitative case study approach. In the 34 case studies reviewed, three described a theoretical framework informed by Stake (1995), two by Yin (2009), and three provided a mixed framework informed by various authors, which might have included both Yin and Stake. Few studies described their case study design, or included a rationale that explained why they excluded or added further procedures, and whether this was to enhance the study design, or to better suit the research question. In 26 of the studies no reference was provided to principal case study authors. From reviewing the description of methods, few authors provided a description or justification of case study methodology that demonstrated how their study was informed by the methodological literature that exists on this approach.

The methodological descriptions of each study were reviewed using the adapted criteria, and the following issues were identified: case study methodology or method; case of something particular and case selection; contextually bound case study; researcher and case interactions and triangulation; and, study design inconsistent with methodology. An outline of how the issues were developed from the critical review is provided, followed by a discussion of how these relate to the current methodological literature.

### Case study methodology or method

A third of the case studies reviewed appeared to use a case report method, not case study methodology as described by principal authors (Creswell, 2013b; Merriam, 2009; Stake, 1995; Yin, 2009). Case studies were identified as a case report because of missing methodological detail and by review of the study aims and purpose. These reports presented data for small samples of no more than three people, places or phenomenon. Four studies, or “case reports” were single cases selected retrospectively from larger studies (Bronken, Kirkevold, Martinsen, & Kvigne, 2012; Coltart & Henwood, 2012; Hooghe, Neimeyer, & Rober, 2012; Roscigno et al., 2012). Case reports were not a case *of* something, instead were a case demonstration or an example presented in a report. These reports presented outcomes, and reported on how the case could be generalized. Descriptions focussed on the phenomena, rather than the case itself, and did not appear to study the case in its entirety.

Case reports had minimal in-text references to case study methodology, and were informed by other qualitative traditions or secondary sources (Adamson & Holloway, 2012; Buzzanell & D'Enbeau, 2009; Nagar-Ron & Motzafi-Haller, 2011). This does not suggest that case study methodology cannot be multimethod, however, methodology should be consistent in design, be clearly described (Meyer, 2001; Stake, 1995), and maintain focus on the case (Creswell, 2013b).

To demonstrate how case reports were identified, three examples are provided. The first, Yeh (2013) described their study as, “the examination of the emergence of vegetarianism in Victorian England serves as a case study to reveal the relationships between boundaries and entities” (p. 306). The findings were a historical case report, which resulted from an ethnographic study of vegetarianism. Cunsolo Willox, Harper, Edge, ‘My Word’: Storytelling and Digital Media Lab, and Rigolet Inuit Community Government (2013) used “a case study that illustrates the usage of digital storytelling within an Inuit community” (p. 130). This case study reported how digital storytelling can be used with indigenous communities as a participatory method to illuminate the benefits of this method for other studies. This “case study was conducted in the Inuit community” but did not include the Inuit community in case analysis (Cunsolo Willox et al., 2013, p. 130). Bronken et al. (2012) provided a single case report to demonstrate issues observed in a larger clinical study of aphasia and stroke, without adequate case description or analysis.

### Case study of something particular and case selection

Case selection is a precursor to case analysis, which needs to be presented as a convincing argument (Merriam, 2009). Descriptions of the case were often not adequate to ascertain why the case was selected, or whether it was a particular exemplar or outlier (Thomas, 2011). In a number of case studies in the health and social science categories, it was not explicit whether the case was of something particular, or peculiar to their discipline or field (Adamson & Holloway, 2012; Bronken et al., 2012; Colón-Emeric et al., 2010; Jackson, Botelho, Welch, Joseph, & Tennstedt, 2012; Mawn et al., 2010; Snyder-Young, 2011). There were exceptions in the methods category (Table III), where cases were selected by researchers to report on a new or innovative method. The cases emerged through heuristic study, and were reported to be particular, relative to the existing methods literature (Ajodhia-Andrews & Berman, 2009; Buckley & Waring, 2013; Cunsolo Willox et al., 2013; De Haene, Grietens, & Verschueren, 2010; Gratton & O'Donnell, 2011; Sumsion, 2013; Wimpenny & Savin-Baden, 2012).

Case selection processes were sometimes insufficient to understand why the case was selected from the global population of cases, or what study of this case would contribute to knowledge as compared with other possible cases (Adamson & Holloway, 2012; Bronken et al., 2012; Colón-Emeric et al., 2010; Jackson et al., 2012; Mawn et al., 2010). In two studies, local cases were selected (Barone, 2010; Fourie & Theron, 2012) because the researcher was familiar with and had access to the case. Possible limitations of a convenience sample were not acknowledged. Purposeful sampling was used to recruit participants *within* the case of one study, but not of the case itself (Gallagher et al., 2013). Random sampling was completed for case selection in two studies (Colón-Emeric et al., 2010; Jackson et al., 2012), which has limited meaning in interpretive qualitative research.

To demonstrate how researchers provided a good justification for the selection of case study approaches, four examples are provided. The first, cases of residential care homes, were selected because of reported occurrences of mistreatment, which included residents being locked in rooms at night (Rytterström, Unosson, & Arman, 2013). Roscigno et al. (2012) selected cases of parents who were admitted for early hospitalization in neonatal intensive care with a threatened preterm delivery before 26 weeks. Hooghe et al. (2012) used random sampling to select 20 couples that had experienced the death of a child; however, the case study was of one couple and a particular metaphor described only by them. The final example, Coltart and Henwood (2012), provided a detailed account of how they selected two cases from a sample of 46 fathers based on personal characteristics and beliefs. They described how the analysis of the two cases would contribute to their larger study on first time fathers and parenting.

Box 1 **Article synopsis of case study research using Stake's tradition**

Ledderer (2011) used a qualitative case study research design, informed by modern ethnography. The study is bounded to 10 general practice clinics in Denmark, who had received federal funding to implement preventative care services based on a Motivational Interviewing intervention. The researcher question focussed on “why is it so difficult to create change in medical practice?” (Ledderer, 2011, p. 27). The study context was adequately described, providing detail on the general practitioner (GP) clinics and relevant political and economic influences. Methodological decisions are described in first person narrative, providing insight on researcher perspectives and interaction with the case. Forty-four interviews were conducted, which focussed on *how* GPs conducted consultations, and the form, nature and content, rather than asking their opinion or experience (Ledderer, 2011, p. 30). The duration and intensity of researcher immersion in the case enhanced depth of description and trustworthiness of study findings. Analysis was consistent with Stake's tradition, and the researcher provided examples of inquiry techniques used to challenge assumptions about emerging themes. Several other seminal qualitative works were cited. The themes and typology constructed are rich in narrative data and storytelling by clinic staff, demonstrating individual clinic experiences as well as shared meanings and understandings about changing from a biomedical to psychological approach to preventative health intervention. Conclusions make note of social and cultural meanings and lessons learned, which might not have been uncovered using a different methodology.

### Contextually bound case study

The limits or boundaries of the case are a defining factor of case study methodology (Merriam, 2009; Ragin & Becker, 1992; Stake, 1995; Yin, 2009). Adequate contextual description is required to understand the setting or context in which the case is revealed. In the health category, case studies were used to illustrate a clinical phenomenon or issue such as compliance and health behaviour (Colón-Emeric et al., 2010; D'Enbeau, Buzzanell, & Duckworth, 2010; Gallagher et al., 2013; Hooghe et al., 2012; Jackson et al., 2012; Roscigno et al., 2012). In these case studies, contextual boundaries, such as physical and institutional descriptions, were not sufficient to understand the case as a holistic system, for example, the general practitioner (GP) clinic in Gallagher et al. (2013), or the nursing home in Colón-Emeric et al. (2010). Similarly, in the social science and methods categories, attention was paid to some components of the case context, but not others, missing important information required to understand the case as a holistic system (Alexander, Moreira, & Kumar, 2012; Buzzanell & D'Enbeau, 2009; Nairn & Panelli, 2009; Wimpenny & Savin-Baden, 2012).

In two studies, vicarious experience or vignettes (Nairn & Panelli, 2009) and images (Jorrín-Abellán et al., 2008) were effective to support description of context, and might have been a useful addition for other case studies. Missing contextual boundaries suggests that the case might not be adequately defined. Additional information, such as the physical, institutional, political, and community context, would improve understanding of the case (Stake, 1998). In Boxes 1 and 2, we present brief synopses of two studies that were reviewed, which demonstrated a well bounded case. In Box 1, Ledderer (2011) used a qualitative case study design informed by Stake's tradition. In Box 2, Gillard, Witt, and Watts (2011) were informed by Yin's tradition. By providing a brief outline of the case studies in Boxes 1 and 2, we demonstrate how effective case boundaries can be constructed and reported, which may be of particular interest to prospective case study researchers.

### Researcher and case interactions and triangulation

Researcher and case interactions and transactions are a defining feature of case study methodology (Stake, 1995). Narrative stories, vignettes, and thick description are used to provoke vicarious experience and a sense of being there with the researcher in their interaction with the case. Few of the case studies reviewed provided details of the researcher's relationship with the case, researcher–case interactions, and how these influenced the development of the case study (Buzzanell & D'Enbeau, 2009; D'Enbeau et al., 2010; Gallagher et al., 2013; Gillard et al., 2011; Ledderer, 2011; Nagar-Ron & Motzafi-Haller, 2011). The role and position of the researcher needed to be self-examined and understood by readers, to understand how this influenced interactions with participants, and to determine what triangulation is needed (Merriam, 2009; Stake, 1995).

Gillard et al. (2011) provided a good example of triangulation, comparing data sources in a table (p. 1513). Triangulation of sources was used to reveal as much depth as possible in the study by Nagar-Ron and Motzafi-Haller (2011), while also enhancing confirmation validity. There were several case studies that would have benefited from improved range and use of data sources, and descriptions of researcher–case interactions (Ajodhia-Andrews & Berman, 2009; Bronken et al., 2012; Fincham, Scourfield, & Langer, 2008; Fourie & Theron, 2012; Hooghe et al., 2012; Snyder-Young, 2011; Yeh, 2013).

Box 2 **Article synopsis of case study research using Yin's tradition**

Gillard et al. (2011) study of camps for adolescents living with HIV/AIDs provided a good example of Yin's interpretive case study approach. The context of the case is bounded by the three summer camps of which the researchers had prior professional involvement. A case study protocol was developed that used multiple methods to gather information at three data collection points coinciding with three youth camps (Teen Forum, Discover Camp, and Camp Strong). Gillard and colleagues followed Yin's (2009) principles, using a consistent data protocol that enhanced cross-case analysis. Data described the young people, the camp physical environment, camp schedule, objectives and outcomes, and the staff of three youth camps. The findings provided a detailed description of the context, with less detail of individual participants, including insight into researcher's interpretations and methodological decisions throughout the data collection and analysis process. Findings provided the reader with a sense of “being there,” and are discovered through constant comparison of the case with the research issues; the case is the unit of analysis. There is evidence of researcher immersion in the case, and Gillard reports spending significant time in the field in a naturalistic and integrated youth mentor role.

This case study is not intended to have a significant impact on broader health policy, although does have implications for health professionals working with adolescents. Study conclusions will inform future camps for young people with chronic disease, and practitioners are able to compare similarities between this case and their own practice (for knowledge translation). No limitations of this article were reported. Limitations related to publication of this case study were that it was 20 pages long and used three tables to provide sufficient description of the camp and program components, and relationships with the research issue.

### Study design inconsistent with methodology

Good, rigorous case studies require a strong methodological justification (Meyer, 2001) and a logical and coherent argument that defines paradigm, methodological position, and selection of study methods (Denzin & Lincoln, 2011b). Methodological justification was insufficient in several of the studies reviewed (Barone, 2010; Bronken et al., 2012; Hooghe et al., 2012; Mawn et al., 2010; Roscigno et al., 2012; Yeh, 2013). This was judged by the absence, or inadequate or inconsistent reference to case study methodology in-text.

In six studies, the methodological justification provided did not relate to case study. There were common issues identified. Secondary sources were used as primary methodological references indicating that study design might not have been theoretically sound (Colón-Emeric et al., 2010; Coltart & Henwood, 2012; Roscigno et al., 2012; Snyder-Young, 2011). Authors and sources cited in methodological descriptions were inconsistent with the actual study design and practices used (Fourie & Theron, 2012; Hooghe et al., 2012; Jorrín-Abellán et al., 2008; Mawn et al., 2010; Rytterström et al., 2013; Wimpenny & Savin-Baden, 2012). This occurred when researchers cited Stake or Yin, or both (Mawn et al., 2010; Rytterström et al., 2013), although did not follow their paradigmatic or methodological approach. In 26 studies there were no citations for a case study methodological approach.

## Discussion

The findings of this study have highlighted a number of issues for researchers. A considerable number of case studies reviewed were missing key elements that define qualitative case study methodology and the tradition cited. A significant number of studies did not provide a clear methodological description or justification relevant to case study. Case studies in health and social sciences did not provide sufficient information for the reader to understand case selection, and why this case was chosen above others. The context of the cases were not described in adequate detail to understand all relevant elements of the case context, which indicated that cases may have not been contextually bounded. There were inconsistencies between reported methodology, study design, and paradigmatic approach in case studies reviewed, which made it difficult to understand the study methodology and theoretical foundations. These issues have implications for methodological integrity and honesty when reporting study design, which are values of the qualitative research tradition and are ethical requirements (Wager & Kleinert, 2010a). Poorly described methodological descriptions may lead the reader to misinterpret or discredit study findings, which limits the impact of the study, and, as a collective, hinders advancements in the broader qualitative research field.

The issues highlighted in our review build on current debates in the case study literature, and queries about the value of this methodology. Case study research can be situated within different paradigms or designed with an array of methods. In order to maintain the creativity and flexibility that is valued in this methodology, clearer descriptions of paradigm and theoretical position and methods should be provided so that study findings are not undervalued or discredited. Case study research is an interdisciplinary practice, which means that clear methodological descriptions might be more important for this approach than other methodologies that are predominantly driven by fewer disciplines (Creswell, 2013b).

Authors frequently omit elements of methodologies and include others to strengthen study design, and we do not propose a rigid or purist ideology in this paper. On the contrary, we encourage new ideas about using case study, together with adequate reporting, which will advance the value and practice of case study. The implications of unclear methodological descriptions in the studies reviewed were that study design appeared to be inconsistent with reported methodology, and key elements required for making judgements of rigour were missing. It was not clear whether the deviations from methodological tradition were made by researchers to strengthen the study design, or because of misinterpretations. Morse (2011) recommended that innovations and deviations from practice are best made by experienced researchers, and that a novice might be unaware of the issues involved with making these changes. To perpetuate the tradition of case study research, applications in the published literature should have consistencies with traditional methodological constructions, and deviations should be described with a rationale that is inherent in study conduct and findings. Providing methodological descriptions that demonstrate a strong theoretical foundation and coherent study design will add credibility to the study, while ensuring the intrinsic meaning of case study is maintained.

The value of this review is that it contributes to discussion of whether case study is a methodology or method. We propose possible reasons why researchers might make this misinterpretation. Researchers may interchange the terms methods and methodology, and conduct research without adequate attention to epistemology and historical tradition (Carter & Little, 2007; Sandelowski, 2010). If the rich meaning that naming a qualitative methodology brings to the study is not recognized, a case study might appear to be inconsistent with the traditional approaches described by principal authors (Creswell, 2013a; Merriam, 2009; Stake, 1995; Yin, 2009). If case studies are not methodologically and theoretically situated, then they might appear to be a case report.

Case reports are promoted by university and medical journals as a method of reporting on medical or scientific cases; guidelines for case reports are publicly available on websites (http://www.hopkinsmedicine.org/institutional_review_board/guidelines_policies/guidelines/case_report.html). The various case report guidelines provide a general criteria for case reports, which describes that this form of report does not meet the criteria of research, is used for retrospective analysis of up to three clinical cases, and is primarily illustrative and for educational purposes. Case reports can be published in academic journals, but do not require approval from a human research ethics committee. Traditionally, case reports describe a single case, to explain how and what occurred in a selected setting, for example, to illustrate a new phenomenon that has emerged from a larger study. A case report is not necessarily particular or the study of a case in its entirety, and the larger study would usually be guided by a different research methodology.

This description of a case report is similar to what was provided in some studies reviewed. This form of report lacks methodological grounding and qualities of research rigour. The case report has publication value in demonstrating an example and for dissemination of knowledge (Flanagan, 1999). However, case reports have different meaning and purpose to case study, which needs to be distinguished. Findings of our review suggest that the medical understanding of a case report has been confused with qualitative case study approaches.

In this review, a number of case studies did not have methodological descriptions that included key characteristics of case study listed in the adapted criteria, and several issues have been discussed. There have been calls for improvements in publication quality of qualitative research (Morse, 2011), and for improvements in peer review of submitted manuscripts (Carter & Little, 2007; Jasper, Vaismoradi, Bondas, & Turunen, 2013). The challenging nature of editor and reviewers responsibilities are acknowledged in the literature (Hames, 2013; Wager & Kleinert, 2010b); however, review of case study methodology should be prioritized because of disputes on methodological value.

Authors using case study approaches are recommended to describe their theoretical framework and methods clearly, and to seek and follow specialist methodological advice when needed (Wager & Kleinert, 2010a). Adequate page space for case study description would contribute to better publications (Gillard et al., 2011). Capitalizing on the ability to publish complementary resources should be considered.

### Limitations of the review

There is a level of subjectivity involved in this type of review and this should be considered when interpreting study findings. Qualitative methods journals were selected because the aims and scope of these journals are to publish studies that contribute to methodological discussion and development of qualitative research. Generalist health and social science journals were excluded that might have contained good quality case studies. Journals in business or education were also excluded, although a review of case studies in international business journals has been published elsewhere (Piekkari et al., 2009).

The criteria used to assess the quality of the case studies were a set of qualitative indicators. A numerical or ranking system might have resulted in different results. Stake's (1995) criteria have been referenced elsewhere, and was deemed the best available (Creswell, 2013b; Crowe et al., 2011). Not all qualitative studies are reported in a consistent way and some authors choose to report findings in a narrative form in comparison to a typical biomedical report style (Sandelowski & Barroso, 2002), if misinterpretations were made this may have affected the review.

## Conclusion

Case study research is an increasingly popular approach among qualitative researchers, which provides methodological flexibility through the incorporation of different paradigmatic positions, study designs, and methods. However, whereas flexibility can be an advantage, a myriad of different interpretations has resulted in critics questioning the use of case study as a methodology. Using an adaptation of established criteria, we aimed to identify and assess the methodological descriptions of case studies in high impact, qualitative methods journals. Few articles were identified that applied qualitative case study approaches as described by experts in case study design. There were inconsistencies in methodology and study design, which indicated that researchers were confused whether case study was a methodology or a method. Commonly, there appeared to be confusion between case studies and case reports. Without clear understanding and application of the principles and key elements of case study methodology, there is a risk that the flexibility of the approach will result in haphazard reporting, and will limit its global application as a valuable, theoretically supported methodology that can be rigorously applied across disciplines and fields.
